# Neonatal outcomes according to different degrees of maternal morbidity: cross-sectional evidence from the Perinatal Information System (SIP) of the CLAP network

**DOI:** 10.1080/16549716.2023.2269736

**Published:** 2023-10-27

**Authors:** Mercedes Colomar, Bremen de Mucio, Claudio Sosa, Rodolfo Gomez, Luis Mainero, Renato T. Souza, Maria L. Costa, Adriana G. Luz, Maria H. Sousa, Carmen M. Cruz, Luz M. Chevez, Rita Lopez, Gema Carrillo, Ulises Rizo, Erika E. Saint Hillaire, William E. Arriaga, Rosa M. Guadalupe, Carlos Ochoa, Freddy Gonzalez, Rigoberto Castro, Allan Stefan, Amanda Moreno, Suzanne J. Serruya, José G. Cecatti

**Affiliations:** a Department of Research, Latin American Center for Perinatology (CLP-PAHO), Montevideo, Uruguay; b Department of Obstetrics and Gynecology, University of Campinas, Campinas, Brazil; c Department of Statistics, Jundiaí School of Medicine - HU/FMJ, Jundiaí, Brazil; d Department of Obstetrics, Hospital Berta Calderon Roque, Managua, Nicaragua; e Department of Obstetrics, Hospital España, Chinandega, Nicaragua; f Department of Obstetrics, Hospital San Lorenzo de Los Mina, Santo Domingo, Dominican Republic; g Department of Obstetrics, Hospital Regional de Ocidente, Quetzaltenango, Guatemala; h Hospital San Felipe, Tegucigalpa, Honduras; i Department of Obstetrics, Hospital Roberto Suazo Cordova, La Paz, Honduras; j Department of Obstetrics, Hospital Leonardo Martinez Valenzuela, San Pedro Sula, Honduras; k Department of Obstetrics, Hospital Boliviano Japones, La Paz, Bolivia

**Keywords:** Neonatal morbidity, neonatal mortality, maternal morbidity, maternal near miss, Latin America

## Abstract

**Background:**

The burden of maternal morbidity in neonatal outcomes can vary with the adequacy of healthcare provision and tool implementation to improve monitoring. Such information is lacking in Latin American countries, where the decrease in severe maternal morbidity and maternal death remains challenging.

**Objectives:**

To determine neonatal outcomes according to maternal characteristics, including different degrees of maternal morbidity in Latin American health facilities.

**Methods:**

This is a secondary cross-sectional analysis of the Perinatal Information System (SIP) database from eight health facilities in five Latin American and Caribbean countries. Participants were all women delivering from August 2018 to June 2021, excluding cases of abortion, multiple pregnancies and missing information on perinatal outcomes. As primary and secondary outcome measures, neonatal near miss and neonatal death were measured according to maternal/pregnancy characteristics and degrees of maternal morbidity. Estimated adjusted prevalence ratios (PRadj) with their respective 95% CIs were reported.

**Results:**

In total 85,863 live births were included, with 1,250 neonatal near miss (NNM) cases and 695 identified neonatal deaths. NNM and neonatal mortality ratios were 14.6 and 8.1 per 1,000 live births, respectively. Conditions independently associated with a NNM or neonatal death were the need for neonatal resuscitation (PR_adj_ 16.73, 95% CI [13.29–21.05]), being single (PR_adj_ 1.45, 95% CI [1.32–1.59]), maternal near miss or death (PR_adj_ 1.64, 95% CI [1.14–2.37]), preeclampsia (PR_adj_ 3.02, 95% CI [1.70–5.35]), eclampsia/HELPP (PR_adj_ 1.50, 95% CI [1.16–1.94]), maternal age (years) (PR_adj_ 1.01, 95% CI [<1.01–1.02]), major congenital anomalies (PR_adj_ 3.21, 95% CI [1.43–7.23]), diabetes (PR_adj_ 1.49, 95% CI [1.11–1.98]) and cardiac disease (PR_adj_ 1.65, 95% CI [1.14–2.37]).

**Conclusion:**

Maternal morbidity leads to worse neonatal outcomes, especially in women suffering maternal near miss or death. Based on SIP/PAHO database all these indicators may be helpful for routine situation monitoring in Latin America with the purpose of policy changes and improvement of maternal and neonatal health.

## Introduction

The concept of maternal near miss, defined as women who nearly died due to pregnancy complications, can be useful to inform policies that help prevent conditions related to maternal adverse outcomes and death [1]. It is often considered a ‘great save’ when the woman survives a severe complication. A systematic review of 35 studies (over 38 million women) evaluated the impact of severe maternal morbidity (SMM) on perinatal outcomes in high-income countries [2]. Overall, women with SMM were four times more likely to experience stillbirth or neonatal death (OR 3.98, 95% CI [3.12–7.60] and OR 3.98, 95% CI [2.44–6.47]), respectively. A prospective cohort study in eastern Ethiopia showed that 32% of perinatal death occurred in women with maternal near miss (MNM). The perinatal mortality ratio was four times higher in women with MNM [3]. In developing countries, the burden of perinatal deaths in cases of maternal morbidity remains uncertain, albeit crucial not only for improving maternal health but also ameliorating its consequences on perinatal outcomes.

Although neonatal mortality rates have decreased in the last decades from 22.5 to 9.1 per 1,000LB in Latin America and the Caribbean [4], significant disparities in perinatal outcomes have been reported among different Latin American and Caribbean countries. Haiti, Guatemala, Dominican Republic and Honduras have the highest neonatal mortality rates (per 1,000 live births) in the region (31.7, 17.5, 24.8, and 16.5, respectively) [5]. In addition, reduction rate in mortality dropped from 4% to 2% approximately [4].

Inaccurate estimates of indicators related to perinatal health have been recognised as a constraint to reducing adverse perinatal outcomes globally. The lack of precise information about the repercussions of maternal morbidity on neonatal outcomes may be attributed to 1) poor identification and registration of maternal morbidity according to standardised definitions; 2) significant limitations of both qualified personnel and well-resourced health facilities; 3) official reports mostly based on estimates derived from small arbitrary and limited epidemiological studies. Civil registration systems in some places are still incomplete and non-reliable, and death certificates are sometimes not requested for stillbirths.

The Latin American Center of Perinatology (CLAP) developed a web-based platform for the surveillance of maternal and neonatal health-related issues called SIP. CLAP also coordinates a Network of sentinel centres (Red CLAP or CLAP Network) in Latin American and Caribbean countries, which monthly report their data to CLAP. All these centres use the Perinatal Information System (Spanish acronym SIP), currently as a common electronic data collection system. SIP was conceived mainly for clinical and surveillance purposes. However, its database has already been used to provide information on maternal outcomes for several studies [5–8]. SIP forms cover information on demographic characteristics, obstetrical information, childbirth data and neonatal and maternal outcomes. SIP can potentially provide a resourceful solution for monitoring health indicators such as conditions associated with neonatal adverse outcomes.

In the current analysis, we aimed to determine adverse neonatal outcomes, according to maternal characteristics, including different degrees of maternal morbidities, based on evidence from the Perinatal Information System (SIP) of five Latin American countries.

## Methods

This is a secondary cross-sectional analysis of the SIP from the Red CLAP Network of sentinel centres. Analysis included data retrieved from the SIP database of all women who gave birth from August 2018 to June 2021 in eight health facilities from Bolivia, Dominican Republic, Guatemala, Honduras and Nicaragua. Data were extracted from the SIP server and considered for analysis after a data consistency check. Records of women with a history of abortion, multiple pregnancies and missing information for perinatal outcomes (missing data related to foetal/neonatal mortality and neonatal near miss) were excluded. These institutions selected for this study were those who implemented the most recent version of the SIP launched in 2018/the new version was updated with a module containing variables related to the degrees of maternal morbidity (potentially life-threatening conditions and maternal near miss). The eight participating health facilities are referral obstetric units covering distinct settings in their districts and countries. Detailed information on their facility capacity and the setting’s Human Development Index is provided in the Supplementary Material (Tables S1 and S2). The characteristics of the health unit was reported according to the Facility Capacity index (FCI), a tool that comprises 34 factors related to human and equipment resources at the facility level [9]. It is usually used to enable accurate management within the institution, to provide a benchmark and compare the relative condition among facilities, considering factors related to human and equipment resources [9–11].

The SIP program is a web-based platform implemented, for more than 40 years, in Latin American and Caribbean countries as part of the Pan American Health Organization – PAHO’s tool kit to improve the quality of care for mothers and newborns [12]. The system provides a standardised electronic record for maternal/neonatal data, which is a purposeful tool for either clinical, management, surveillance, policy-making and research. The system has made it possible to check and monitor the implementation of evidence-based practices, unify and standardise data collection, provide reliable statistics and inter-institution communication, audit to improve and assess quality of care, and conduct epidemiological studies.

Maternal/pregnancy characteristics were assessed as independent variables associated with adverse neonatal outcomes. Independent variables included degrees of maternal morbidities according to WHO definition and criteria [1], as follows (PLTC: potentially life-threatening condition; SMO: severe maternal outcome (MNM+MD); MNM: maternal near miss; MD: maternal death), maternal age, ethnicity, literacy, marital status, parity, planned pregnancy, contraceptive use, medical history, number of antenatal care visits, habits and lifestyle (smoking, alcohol), obstetric history, and onset of labour. Degrees of maternal morbidity were based on the concept of a continuum of maternal morbidity and maternal near miss [1]; the use of the concept was previously validated for SIP database [13]. The criteria for PLTC, MNM and SMO as defined by the WHO are provided in detail in Supplementary Material (Supplementary Material; Box 1). Neonatal outcomes were estimated according to degrees of maternal morbidity, including neonatal near-miss (NNM), neonatal deaths (ND), and severe neonatal outcomes (NNM or ND). Neonatal near miss was defined as birthweight <1750 g, 5-minute Apgar <7 or gestational age at delivery <33 weeks [14]. The proportion of neonatal near miss/neonatal mortality cases, neonatal near miss ratio (NNMR: number of NNM cases per 1,000 live births), and neonatal mortality rate (NMR: number of neonatal deaths per 1,000 live births) were calculated. In addition, mode of delivery, health professional responsible for childbirth care, gestational age at birth, birthweight, 5-minute Apgar score, neonatal resuscitation and congenital anomalies were assessed as confounders for neonatal outcome.

On bivariate analyses, the risk of neonatal near miss and neonatal death was estimated according to characteristics of maternal, pregnancy and perinatal care, including degrees of maternal morbidity. Prevalence ratios (PR) with their 95% confidence intervals (CI) were reported for each predictor. Then, a backward stepwise logistic regression analysis was performed to assess conditions independently associated with severe neonatal outcomes (neonatal near miss or neonatal death). Variables related to characteristics of maternal, pregnancy and perinatal care were all included in the model, adjusting for significant predictors and cluster design (eight facilities, i.e. the PSU was the only survey design characteristic in this study). The models were selected by Bayesian information criteria (BIC). The Huber/White variance estimator, with clustered data, was used, which allow for intragroup correlation (cluster). The variable selection was performed manually, starting with those with higher p-values until those with p-values <0.05. All maternal variables were included in the first model. We reported the estimated adjusted prevalence ratios (PRadj) with their respective 95% CIs. The software Stata version 7.0 (StataCorp, College Station, TX, USA) was used for data analysis.

### Ethical aspects

This analysis is part of the ‘Study on the incidence of severe maternal morbidity and mortality in maternities from the Red-CLAP in Latin America and the Caribbean’, approved by the Research Ethics Committee (REC) from the Pan American Health Organization (PAHO) on 17 August 2018 (PAHOERC Ref. No: PAHO-2018-04-0025). Details of the study protocol were previously published elsewhere [15].

## Results

**Figure 1.**
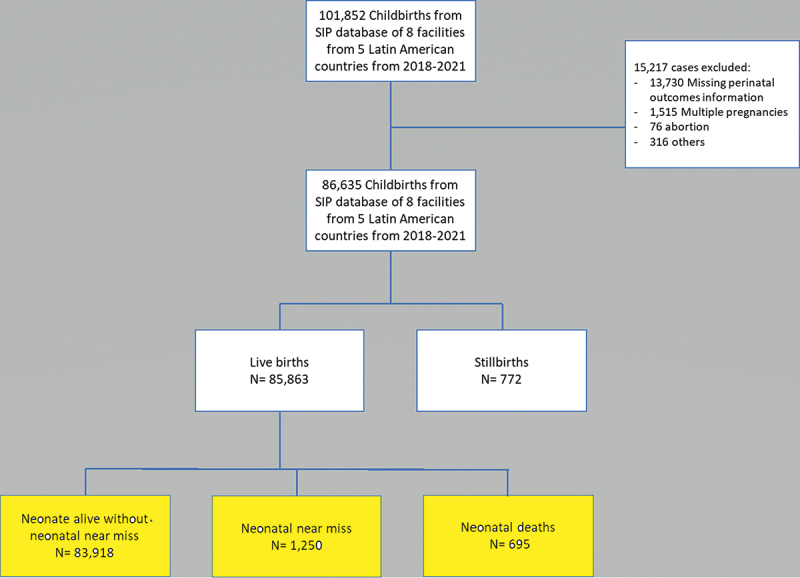
Flowchart of participants in the study according to perinatal outcomes (SIP-PAHO, 2018–2021).

In total, 101,852 childbirths composed the SIP database of eight facilities in five Latin American countries from 2018 to 2021. After excluding cases with missing data on perinatal outcomes (*n* = 13,730), multiple pregnancies (*n* = 1,515), abortion (*n* = 76) and other minor reasons (*n* = 316; e.g. outliers for gestational age at delivery – over 42 weeks of gestation), 86635 cases were considered for analysis, with 85,863 live births (LB) and 772 stillbirths. Figure 1 shows the flowchart of cases considered for analysis. We included 83,918 cases whose neonates were alive and had no near miss events, 1,250 (1.5%) neonatal near miss cases and 695 (0.8%) neonatal deaths. The neonatal near miss rate (NNMR) was 14.6/1,000LB, and neonatal mortality rate (NMR) was 8.1/1,000LB.

On bivariate analysis, risks for neonatal near miss and neonatal death were assessed according to maternal and obstetric characteristics (Table 1). Conditions associated with neonatal near miss were maternal age between 30 and 34 years (PR 1.32; 95% CI [1.03–1.71]), maternal age ≥35 years (PR 1.63; 95% CI [1.28–2.08]), indigenous ethnicity (PR 2.06; 95% CI [1.25–3.39]), not having a partner (PR 1.57; 95% CI [1.23–2.00]), and elective C-section (PR 1.91; 95% CI [1.27–2.87]). Multiparous women were less likely to have neonatal near miss (PR 0.84; 95% CI [0.72–0.96]). Conditions associated with neonatal death were maternal age between 30 and 34 years (PR 1.40; 95% CI [1.25–1.58]) and ≥35 years (PR 1.81; 95% CI [1.21–2.70]). On contrary for NNM, neonatal death was less likely to occur for babies born to indigenous women (PR 0.84; 95% CI [0.72–0.96]).

**Table 1.** Crude estimated risks (PR) of neonatal near miss and neonatal death according to some maternal and obstetric characteristics. SIP/PAHO 2018–2021.

	Alive without severe complication	Neonatal near miss*		Neonatal death	
Characteristics	N (%)	N (%)	PR (95%CI)	N (%)	PR (95%CI)
**Maternal age (years)a**					
<20	21269 (25.6)	308 (25.0)	1.15 (0.95–1.40)	179 (26.2)	1.20 (0.91–1.58)
20–24	25945 (31.3)	338 (27.5)	Ref.	182 (26.6)	Ref.
25–29	18830 (22.7)	265 (21.5)	1.08 (0.89–1.31)	137 (20.0)	1.04 (0.85–1.26)
30–34	10634 (12.8)	184 (14.9)	**1.32(1.03–1.71)**	105 (15.4)	**1.40 (1.25–1.58)**
≥35	6344 (7.6)	136 (11.0)	**1.63 (1.28–2.08)**	81 (11.8)	**1.81 (1.21–2.70)**
**Ethnicity/Skin colorb**					
White	529 (0.6)	13 (1.1)	1.78 (0.92–3.42)	1 (0.1)	0.22 (0.01–4.48)
Mixed	76026 (91.8)	1041 (84.6)	Ref.	645 (96.0)	Ref.
Indigenous	5796 (7.0)	166 (13.5)	**2.06 (1.25–3.39)**	24 (3.6)	**0.49 (0.28–0.86)**
Others	432 (0.5)	10 (0.8)	1.67 (0.76–3.71)	2 (0.3)	0.55 (0.15–1.95)
**Literacyc**					
No or primary	29536 (35.8)	486 (39.5)	1.17 (0.79–1.73)	231 (34.3)	0.93 (0.70–1.25)
Secondary or University	52926 (64.2)	745 (60.5)	Ref.	443 (65.7)	Ref.
**Marital statusd**					
Married/stable partner	76998 (94.5)	1113 (91.5)	Ref.	619 (93.4)	Ref.
Single/Other	4499 (4.5)	103 (8.5)	**1.57 (1.23–2.00)**	44 (6.6)	1.21 (0.73–2.01)
**Paritye**					
Nullipara	34792 (41.9)	574 (46.4)	Ref.	296 (43.5)	Ref.
Multipara	48235 (58.1)	663 (53.6)	**0.84 (0.72–0.96)**	384 (56.5)	0.94 (0.77–1.14)
Previous C-section*f	12783 (27.5)	199 (30.7)	1.17 (0.92–1.48)	118 (31.6)	1.21 (0.86–1.69)
**Syphilisg**					
Yes	520 (0.7)	7 (0.6)	0.79 (0.25–2.52)	8 (1.2)	1.90 (0.86–4.18)
No	34856 (43.6)	594 (49.7)	Ref.	280 (42.7)	Ref.
Not done	44512 (55.7)	595 (49.7)	0.79 (0.39–1.60)	368 (56.1)	1.03 (0.61–1.72)
**Onset of laborh**					
Spontaneous	59454 (71.8)	745 (60.6)	Ref.	407 (60.1)	Ref.
Induced	5550 (6.7)	54 (4.4)	0.78 (0.28–2.17)	39 (5.8)	1.03 (0.55–1.91)
Elective C-section	17834 (21.5)	431 (35.0)	**1.91 (1.27–2.87)**	231 (34.1)	1.88 (0.90–3.94)
**Total**	83,918	1,250		695	

*NNM/Neonatal Near Miss: birthweight <1750 g or 5-min Apgar < 7 or GA <33 weeks.

PR: Crude prevalence ratio (adjusted for cluster effect). *Only for multiparous woman.

Missing information on a: 926; b:1,178; c: 1,496; d: 2,487; e: 919; f: 1,869; g: 4,123; h: 1,118.

Values in bold mean they are statistically significant.

Table 2 shows the absolute and relative risks for neonatal adverse outcomes according to maternal health conditions. Neonatal near miss was significantly associated with women with diabetes (PR 3.23; 95% CI [2.29–4.55]), chronic hypertension (PR 2.70; 95% CI [1.50–4.87]), preeclampsia (PR 5.14; 95% CI [3.05–8.68]), eclampsia/HELLP (PR 8.81; 95% CI [3.38–22.97]), cardiac disease (PR 3.91; 95% CI [1.70–8.99]), renal disease (PR 3.91; 95% CI [1.70–25.42]), and any medical condition (PR 2.68; 95% CI [1.49–4.82]). Neonatal death was significantly associated with maternal anaemia (PR 2.40; 95% CI [1.61–3.58]), chronic hypertension (PR 3.01; 95% CI [1.78–5.06]), preeclampsia (PR 5.10; 95% CI [3.12–8.32]), eclampsia/HELLP (PR 5.76; 95% CI [1.67–19.92]), cardiac disease (PR 4.68; 95% CI [1.63–13.44]), renal disease (PR 11.85; 95% CI [6.54–21.48]), and any of the above medical conditions (PR 3.45; 95% CI [2.41–4.92]). Degrees of maternal morbidity were also associated with adverse neonatal outcomes. Potentially life-threatening condition (PTLC), maternal near miss (MNM), maternal death (MD) and severe maternal outcome (SMO) were associated with a fivefold, sevenfold, ninefold and sevenfold increase in the risk for neonatal near miss, respectively. The risk for neonatal death was higher in women who had a PTLC (PR 4.60; 95% CI [2.83–7.46]), MNM (PR 10.34; 95% CI [5.64–18.94]), MD (PR 14.17; 95% CI [2.84–70.71]) and SMO (PR 10.95; 95% CI [7.02–17.08]) (Table 3).

**Table 2.** Crude estimated risks (PR) of neonatal near miss and neonatal death according to some maternal health conditions. SIP/PAHO 2018–2021.

	Alive without NNM (*n* = 83,918)	Neonatal near miss* (*n* = 1,250)		Neonatal death (*n* = 695)	
Medical conditions	N (%)	N (%)	PR (95%CI)	N (%)	PR (95%CI)
Anemia^a^	6936 (8.4)	122 (9.9)	1.20 (0.67–2.15)	123 (18.1)	**2.40 (1.61–3.58)**
Diabetes^b^	448 (0.5)	22 (1.8)	**3.23 (2.29–4.55)**	14 (2.1)	**3.80 (1.60–9.05)**
Hypertension^c^	1452 (1.7)	58 (4.7)	**2.70 (1.50–4.87)**	35 (5.1)	**3.01 (1.78–5.06)**
Preeclampsia^d^	3994 (4.8)	266 (21.5)	**5.14 (3.05–8.68)**	142 (20.9)	**5.10 (3.12–8.32)**
Eclampsia/HELLP^e^	615 (0.7)	85 (6.9)	**8.81 (3.38–22.97)**	29 (4.3)	**5.76 (1.67–19.92)**
Cardiac disease^f^	282 (0.3)	17 (1.4)	**3.91 (1.70–8.99)**	11 (1.6)	**4.68 (1.63–13.44)**
Renal disease^g^	76 (0.1)	8 (0.6)	**6.53 (1.67–25.42)**	8 (1.2)	**11.85 (6.54–21.48)**
Other severe medical condition^h^	462 (0.6)	2 (0.2)	0.29 (0.05–1.77)	6 (0.9)	1.59 (0.94–2.70)
Any medical condition^i^	12377 (14.7)	401 (32.1)	**2.68 (1.49–4.82)**	262 (37.7)	**3.45 (2.41–4.92)**
Continuum of Maternal morbidity^j^					
No morbidity	74318 (94.7)	901 (75.5)	Ref.	503 (77.0)	Ref.
PLTC	3796 (4.8)	257 (21.5)	**5.29 (2.69–10.41)**	121 (18.5)	**4.60 (2.83–7.46)**
MNM	308 (0.4)	29 (2.4)	**7.18 (3.62–14.26)**	23 (3.5)	**10.34 (5.64–18.94)**
MD	57 (0.1)	7 (0.6)	**9.13 (2.83–29.48)**	6 (0.9)	**14.17 (2.84–70.71)**
SMO	365 (0.5)	36 (3.0)	**7.49 (3.72–15.11)**	29 (4.4)	**10.95 (7.02–17.08)**

*NNM/Neonatal Near Miss: birthweight <1750 g or 5-min Apgar < 7 or GA <33 weeks. PTLC: Potentially life-threatening condition; MNM: Maternal near-miss; SMO: severe maternal outcome (MNM or maternal death); MD: maternal death.

Missing information for a:942; b: 630; c: 625; d: 784; e: 774; f: 545; g: 544; h: 766; i: 1242; j: 5,537.

Values in bold mean they are statistically significant.

**Table 3.** Crude estimated risks (PR) of neonatal near miss and neonatal death according to some pregnancy outcomes. SIP/PAHO 2018–2020.

	Alive without NNM (*n* = 83,918)	Neonatal near miss* (*n* = 1,250)		Neonatal death (*n* = 695)		
Pregnancy outcomes	N (%)	N (%)	PR (95%CI)	N (%)	PR (95%CI)	p-value#
**Mode of delivery**^a^						.053
C-section	30736 (36.8)	654 (52.6)	**1.90 (1.43–2.53)**	340 (49.1)	**1.64 (1.08–2.51)**	
Spontaneous vaginal	52700 (63.1)	584 (46.9)	Ref.	353 (50.9)	Ref.	
Operative vaginal	93 (0.1)	6 (0.5)	**5.53 (2.68–11.43)**	0 (-)	–	
**Assistance during childbirth**^b^						**<.001**
MD/obstetrician	68912 (84.8)	1121 (90.7)	Ref.	653 (96.0)	Ref.	
Nurse	3017 (3.7)	12 (1.0)	**0.25 (.14–.44)**	4 (0.6)	**0.14 (.07–.29)**	
Only other health professionals	9327 (11.5)	103 (8.3)	0.68 (.24–1.91)	23 (3.4)	0.26 (.07–1.04)	
**Gestational age at birth**^c^						
<32 weeks	116 (0.1)	354 (28.8)	–	312 (46.2)	**27.8 (183.0–4.7)**	
32–36	4637 (5.6)	412 (33.6)	–	154 (22.8)	**11.94 (6.91–2.63)**	
≥37 weeks	77792 (94.2)	462 (31.1)	–	210 (31.1)	Ref.	
**Birth weight**						
<2500 g	5191 (6.2)	881 (70.5)	–	482 (69.4)	**32.07 (19.74–52.11)**	
2500–3999 g	76806 (91.5)	347 (27.8)	–	204 (29.4)	Ref.	
≥4000 g	1921 (2.3)	22 (1.8)	–	9 (1.3)	1.76 (.66–4.69)	
**Congenital anomalies**^d^	560 (0.7)	37 (3.0)	**4.31 (2.36–7.87)**	91 (13.4)	**19.81 (6.56–59.78)**	**<.001**
**5-min Apgar < 7**^e^	18 (<0.1)	377 (30.3)	–	189 (27.3)	**152.9 (76.4–305.8)**	
**Neonatal resuscitation required**^f^	1667 (2.0)	433 (34.8)	**2.95 (17.15–25.60)**	307 (44.5)	**33.29 (17.57–63.07)**	**<.001**

*Neonatal Near Miss: birthweight <1750 g or 5-min Apgar < 7 or GA <33 weeks. #Comparison between neonatal near-miss and neonatal death.

Missing information for: a: 397; b: 2691; c: 1414; d: 510; e: 6; f: 654.

Table 3 shows the risks for neonatal outcomes according to some pregnancy outcomes. Neonates born to low-risk pregnant women assisted solely by nurses during childbirth were less likely to suffer neonatal near miss (PR 0.25; 95% CI [0.14–0.44]) or neonatal death (PR 0.14; 95% CI [0.07–0.29]). Conditions associated with neonatal near miss included having a C-section (PR 1.90; 95% CI [1.43–2.53]) or operative vaginal birth (PR 5.53; 95% CI [2.68–11.43]), congenital anomalies (PR 4.31 95% CI [2.36–7.87]), and the need for neonatal resuscitation (PR 20.95; 95% CI [17.15–25.60]). Conditions associated with neonatal death were as follows: C-section (PR 1.64; 95% CI [1.08–2.51]), gestational age below 32 weeks at birth (PR 270.8; 95% CI [183.0–400.7]) or between 32 and 36 weeks (PR 11.94; 95% CI [6.91–20.63]), birth weight below 2500 g (PR 32.07; 95% CI [19.74–52.11]), congenital anomalies (PR 19.81 95% CI [6.56–59.78]), Apgar score below 7 at 5 minutes (PR 152.9; 95% CI [76.4–305.8]), and neonatal resuscitation (PR 33.29; 95% CI [17.57–63.07]).

Variables independently associated with severe neonatal outcomes (neonatal near miss or death) in Table 4 were neonatal resuscitation (PR_adj_ 16.73; 95% CI [13.29–21.05]), not having a partner (PR_adj_ 1.45; 95% CI [1.32–1.59]), severe maternal outcome (PR_adj_ 1.64; 95% CI [1.14–2.37]), preeclampsia (PR_adj_ 3.02; 95% CI [1.70–5.35]), eclampsia/HELLP (PR_adj_ 1.50; 95% CI [1.16–1.94]), maternal age (years; PR_adj_ 1.01; 95% CI [<1.01–1.02]), major congenital anomalies (PR_adj_ 3.21; 95% CI [1.43–7.23]), diabetes (PR_adj_ 1.49; 95% CI [1.11–1.98]), and maternal cardiac disease (PR_adj_ 1.65; 95% CI [1.14–2.37]). From the 86.635 cases included in the analysis 77,656 remained in the multivariate analysis. Within the 8,979 missing cases, 165 (8.5% of 1.945) were events of interest. Variables with the most missing information were severe maternal outcome (SMO) (67.5%) and marital status (30.3%). Each of the seven variables in the final model presented less than 11.5% of missing.

**Table 4.** Variables independently associated with severe neonatal outcomes (neonatal near miss or neonatal death). SIP/PAHO 2018–2021 [*n* = 77,656].

Variables	PR_adj_	95% CI	p-value
Neonatal resuscitation required (yes)	16.73	13.29–21.05	<0.001
Marital status (single/other)	1.45	1.32–1.59	<0.001
SMO (MNM+MD)	1.64	1.14–2.37	0.001
Preeclampsia	3.02	1.70–5.35	0.003
Eclampsia/HELLP	1.50	1.16–1.94	0.008
Maternal age (years)	1.01	<1.01–1.02	0.009
Major Congenital anomalies	3.21	1.43–7.23	0.011
Diabetes	1.49	1.11–1.98	0.014
Cardiac disease	1.65	1.14–2.37	0.014

PR_adj_ = Prevalence Ratio adjusted for significant predictors and cluster design (eight facilities). CI confidence interval for PR. Predictors entering in the first model: Maternal age (years); Ethnicity (Mixed:0/other:1); Literacy (No or primary:1/Secondary or university:0); Marital status (Married+ stable part:0/Single+other:1); Parity (0/>0:1); Syphilis (Yes:1/No, not done:0); Onset of labor: Spontaneous/Induced+Elective C-section); Anemia (Yes:1/No:0); Diabetes (Yes:1/No:0); Hypertension (Yes:1/No:0); Preeclampsia (Yes:1/No:0); Eclampsia/HELLP (Yes:1/No:0); Cardiac disease (Yes:1/No:0); Renal disease (Yes:1/No:0); Other severe medical condition (Yes:1/No:0); SMO (MNM+MD:1/No morbidity+PLTC:0); Mode of delivery (Spontaneous vaginal:0/C-section+operative vaginal:1); Assistance during childbirth (MD+obstetrician:0/Nurse+only other health professionals:1); Congenital anomalies (‘Major’:1/No+’minor’:0); Neonatal resuscitation required (Yes:1/No:0).

## Discussion

The current study addressed neonatal outcomes, based on different degrees of maternal complications from eight health facilities in five Latin American and Caribbean countries (Bolivia, Guatemala, Honduras, Nicaragua and the Dominican Republic). With increasing maternal morbidity severity, there was a higher risk of worse neonatal outcome, including a higher risk for neonatal near miss and mortality. The association between worse neonatal outcomes and maternal chronic diseases (diabetes, cardiac disease), pregnancy-related complications (preeclampsia, eclampsia/HELLP, major congenital anomalies) and severe maternal morbidity highlights the importance of healthcare quality and coverage in low-resource settings, and the provision of proper antenatal care surveillance.

A previous study using SIP data from 12 Latin American and Caribbean countries (Argentina, Bolivia, Colombia, El Salvador, Ecuador, Guatemala, Guiana, Honduras, Haiti, Nicaragua, Paraguay, and Uruguay) included more than 700,000 childbirths between 2009 and 2012 [12]. Overall, neonatal near miss and neonatal mortality rates were around 51.6 per 1,000LB and 5.85 per 1,000LB, respectively. Neonatal near miss occurred in about one in every seven women who experienced maternal near miss or died. Neonatal mortality occurred in 2–3% of women. Neonatal near miss and neonatal mortality occurred in only 4.9% and 0.6% of neonates born to women with no morbidity. However, that study could not use variables specific for PLTC and MNM classification because this surveillance had not yet been inserted into the SIP database. In our current study, maternal near miss or maternal death resulted in a 7 to 9-fold increased risk for neonatal near miss and a 10 to 14-fold higher risk for neonatal mortality.

The occurrence of neonatal near miss vvaries across different low- and middle-income countries. While we found a prevalence of 1.4% of neonatal near miss, a systematic review conducted in Ethiopia estimated a pooled prevalence of 35.5% of NNM. Another systematic from African countries included eight studies and reported a prevalence of NNM of 30%. The great burden of pregnancy complication and maternal morbidity in the development of adverse neonatal outcomes in low- and middle-income countries has been endorsed by the literature. In Brazil, two nationwide multicenter studies reported that hypertensive disorders, maternal age and severe maternal outcome (maternal near miss or death) were significantly associated with adverse neonatal outcomes, including neonatal near miss [16,17]. Neonates from mother with more than 35 years (1.3-fold) [17], hypertensive disorders (2.4-fold) [17] in pregnancy or maternal near miss or death (1.8-fold) [16] were more likely to experience neonatal near miss. The need for a standard definition for NNM and remarkable differences in the provision of adequate antenatal and intrapartum care might be the reasons for such distinct estimates in the different settings. This indicates the need for a standard definition for NNM and the development of strategies for improving the quality of maternal and neonatal health care, which are some of the core purposes of implementing SIP in Latin American and Caribbean countries [12,13].

A Brazilian cohort study including 311 women with severe maternal morbidity and 323 without SMM addressed neonatal consequences that were associated with SMM [18]. In that study, severe maternal morbidity was shown to be associated with a higher risk of preterm birth (49.0% versus 11.1%, p-value < 0.001), perinatal death (7.4% versus 2.2%, p-value = 0.004), low birth weight (45.8% versus 11.5%, p-value < 0.001) and gestational age below 30 weeks (7.5% versus 0.9%, p-value < 0.001). Furthermore, infants of mothers who experienced SMM were more likely to screen positive for neurodevelopmental impairment according to the Denver II test (RR 1.55 95% CI [1.02–2.36]). Our findings reinforce the importance of providing proper antepartum and intrapartum care for this particular higher-risk population. In addition, early screening for abnormal development should be implemented with adequate follow-up of neonates [19]. Severe neonatal outcome depends not only on the occurrence of maternal complications during pregnancy and intrapartum period but especially a timely identification and management of such complications.

Most Latin American countries have advanced in their epidemiological transition, partly owing to reduced child and infant mortality rates. Numerous initiatives, such as healthcare system reform, conditional cash transfer programmes, and universal basic healthcare coverage, have aimed to enhance maternal and infant health [20]. The SIP, an online standardised platform for perinatal data monitoring and auditing in Latin American and Caribbean nations, serves as a valuable tool for assessing regional healthcare status and guiding maternal and infant health programme priorities. Additionally, it tailors its approach to each country’s specific needs, thereby contributing to the reduction of disparities in regional healthcare system characteristics and outcomes.

The identification or early detection of conditions associated with neonatal near miss or neonatal death can be useful for understanding the burden of maternal complications, setting strategies to improve quality of care and resources based on risk-stratification models to mitigate the impact and reduce consequences, implementing early community-based detection of obstetric complications related to worse neonatal outcomes and improving facility birth outcomes. To achieve this, adequate referral for high-risk women to receive appropriate care is mandatory. A predictive modelling analysis based on a multicenter cohort study of over 480,000 neonates from resource-limited settings (six South Asian, African, and Latin American countries) assessed the predictors for intrapartum stillbirth and neonatal mortality. The study compared models with antenatal and delivery/post-delivery variables [19]. The most accurate predictors included birth weight, antenatal and neonatal support (bag/mask resuscitation, neonatal antibiotics, neonatal hospitalisation), gestational age at delivery and maternal/pregnancy characteristics, e.g. maternal age, parity, haemorrhage, hypertensive disorders, sepsis, education and multiple pregnancies. Models with the best performances were based on post-delivery variables (area under curve ROC of 0.8–0.9). Pre-delivery and antenatal variables had models with AUC ROC of 0.6–0.7 and around 0.7, respectively. In conclusion, prenatal and predelivery data were not sufficient to support strategies for screening, monitoring and referral of women at higher risk for neonatal mortality in that study [20]. In contrast, our study highlights the importance of severe maternal morbidity, showing that maternal chronic diseases and pregnancy-related complications are independently associated with neonatal morbidity and mortality.

The current study utilises the SIP database, a robust facility-based electronic health information system containing perinatal data, as a data source. Such routine facility-based data hold potential for epidemiological studies, health indicator monitoring, and promoting interoperable datasets, facilitating inter-level communication [21–23]. Although significant efforts have been invested in enhancing data coverage, completeness, and quality within the SIP system, limitations persist, such as the insensitivity of certain indicators, reliance on outpatient data (e.g. deliveries outside obstetric units), and variations in data quality linked to local standards and audit frequency [24]. Despite attempts to enhance data quality, 13.4% of cases had to be excluded from the analysis due to missing perinatal outcome information. Additionally, it is crucial to note that the SIP database comprises data from eight facilities across five Latin American and Caribbean countries and does not offer representative coverage of the respective nations or the broader Latin American region. Our study did not account for some factors that may influence perinatal outcomes (neonatal near miss) such as social strata, quality of antenatal care, maternal infections, levels of care in birth hospitals, and substance abuse. We would like to acknowledge that there are different criteria for neonatal near miss, with different the number and ‘nature’ of criterium. Different approaches can result in different estimates of neonatal near miss rates; there are reasonable reasons supporting each choice. The pragmatic criteria used in our study are sensible, especially when applied to live surveillance hospital-based datasets; the information comprising the pragmatic criteria is easily collected and facilitates its reproducibility. A comparative cohort study conducted in 2017 in six health facilities in Brazil concluded that near miss definitions that are exclusively derived from pragmatic criteria can be considered sensible and can effectively serve the purpose of monitoring [25]. A recent literature review described seven different definitions of NNM without any indication of international consensus [26]. Notably, the identification of NNM, particularly in low- and middle-income countries, heavily relied on pragmatic criteria such as birth weight, gestational age, and Apgar score [26]. Therefore, we stand for using a criterion with a higher sensitivity, considering that the purpose is to identify associated factors to support improvements of care to targeted populations (which are at higher risk for NNM). Finally, we would like to acknowledge that the missing cases in the multivariate analysis (8,979 missing cases, which 165 (8.5% of 1.945) were events of interest) can potentially impact the estimation of interactions. The PAHO/CLAP, responsible for promoting, implementing, and surveilling data quality indicators of the platform, has been working on improving data quality and decreasing data missing.

Maternal morbidity significantly impacts neonatal outcomes, particularly in cases of maternal near miss or mortality. Utilising neonatal near miss and neonatal mortality ratios from the SIP/PAHO database can serve as reasonable indicators for routine monitoring in Latin American countries. This monitoring is vital for evaluating healthcare structures, processes, and outcomes related to effective interventions aimed at reducing neonatal mortality [27]. In conclusion, the Perinatal Information System (SIP/PAHO) offers valuable insights into maternal characteristics, varying degrees of maternal morbidity, and their association with severe neonatal outcomes. It can aid in 1) assessing the burden of neonatal near miss in healthcare facilities using standardised criteria, 2) identifying associated factors, 3) promoting improved maternal and neonatal healthcare based on identified risk factors, and ultimately, 4) demonstrating its utility as a resourceful continuous monitoring tool for reducing adverse outcomes.

## Supplementary Material

Supplemental Material

## Data Availability

The property of data used in this manuscript is of each participating country, coordinated by the PAHO-CLAP in Montevideo, Uruguay. The data can be available from there upon a reasonable request.

## Paper context

We assessed the burden of maternal morbidity in neonatal outcomes in Latin American countries, representing an attempt to improve both maternal and neonatal health in the region. More than 87 thousand births from five countries were included with information from an electronic health information system containing perinatal data. With the increasing severity of maternal morbidity, there is a higher risk of poor neonatal outcomes, which can be used as routine monitoring for health status.
