# Supplementary material for: Neonatal outcomes according to different degrees of maternal morbidity: cross-sectional evidence from the Perinatal Information System (SIP) of the CLAP network
Source: Glob Health Action. 2023 Oct 27;16(1):2269736. doi: 10.1080/16549716.2023.2269736 (PMC10795600; doi:10.1080/16549716.2023.2269736)
Supplement: Supplemental Material [file ZGHA_A_2269736_SM4972.docx]

**Supplementary Material**

**Table S1. Facility Capacity Index (FCI)** – Essential/additional provision of care performed by each institution between 2018 and 2021 (“X”=yes).

| **Category** | **Essential/Additional services** | **Center01**  **Nicaragua** | **Center02**  **Nicaragua** | **Center03**  **Bolivia** | **Center04**  **Honduras** | **Center05**  **Honduras** | **Center06**  **Honduras** | **Center07**  **Guatemala** | **Center07**  **Dominican Republic** |
| --- | --- | --- | --- | --- | --- | --- | --- | --- | --- |
| Standard of building / basic services | Electricity, water supply and sewerage system | X | X | X | X | X | X | X | X |
|  | Generator | X | X | X | X | X | X | X | X |
|  | Refrigerator | X | X | X | X | X | X | X | X |
|  | Sterilization | X | X | X | X | X | X | X | X |
|  | Telephone | X | X | X | X | X | X | X | X |
|  | Ambulance | Centralized EMS* | X | X | X | X | X | X | X |
| Medical services | Blood bank | X | X | X | X | X | X | X | X |
|  | Routine screening of donor blood (HIV, Hep B, Syphilis) | X | X | X | X | X | X | X | X |
|  | Biochemical/clinical laboratories | X | X | X | X | X | X | X | X |
|  | Adult intensive care unit | X | X |  |  |  |  | X | X |
|  | Neonatal intensive care unit, or any other newborn care with indicators | X | X | X | X |  |  | X | X |
|  | High risk pregnancy beds | X | X | X |  |  | X | X | X |
| Emergency obstetric services | Administration of parental antibiotics | X | X | X | X | X | X | X | X |
|  | Administration of uterotonics (oxytocin, misoprostol or other uterotonics) | X | X | X | X | X | X | X | X |
|  | Administration o f magnesium sulphate | X | X | X | X | X | X | X | X |
|  | Manual removal of placenta | X | X | X | X | X | X | X | X |
|  | Removal of retained products | X | X | X | X | X | X | X | X |
|  | Vacuum extraction or forceps delivery |  | X | X | X | X | X |  |  |
|  | Blood transfusion | X | X | X | X | X | X | X | X |
|  | Hysterectomy | X | X | X | X | X | X | X | X |
|  | Neonatal Resuscitation | X | X | X | X | X | X | X | X |
| **Category** | **Essential/Additional services** |  |  |  |  |  |  |  |  |
| Laboratory tests | Haemoglobin | X | X | X | X | X | X | X | X |
|  | Platelet count | X | X | X | X | X | X | X | X |
|  | Coagulation tests | X | X | X | X | X | X | X | X |
|  | Bilirubin | X | X | X | X | X | X | X | X |
|  | Lactate | X | X | X | X | X | X | X |  |
|  | Blood gas analysis | X | X | X |  | X |  | X | X |
|  | Creatinine | X | X | X | X | X | X | X | X |
| Hospital practices | WHO/local guidelines currently in use | X | X | X | X | X | X | X | X |
|  | Maternal death review | X | X | X | X | X | X | X |  |
| Hospital Resources | Obstetrician available 24/7, in the facility | X | X | X | X | X | X | X | X |
|  | Obstetrician available 24/7, on call | X | X | X | X | X |  | X | X |
|  | Anaesthesiologist 24/7, in the facility | X | X | X | X | X |  | X | X |
|  | Anaesthesiologist 24/7, on call | X | X | X | X | X |  | X | X |

* Emergency medical services (ambulance center).

**Table S2. Human Development Index** – National and sub-national HDI from the location of each participating health facility

| País | Country  HDI (2019)* | HDI Classification*  (Country) | District | District/Departament  HDI (2019)* | HDI classification*  (District/Departament) |
| --- | --- | --- | --- | --- | --- |
| Center 01 - Nicaragua | 0,660 | Medium | Managua | 0.709 | High |
| Center 02 - Nicaragua | 0,660 | Medium | Chinandega | 0.709 | High |
| Center 03 - Bolivia | 0,718 | High | La Paz | 0.719 | High |
| Center 04 - Honduras | 0,634 | Medium | Cortes | 0.676 | Medium |
| Center 05 - Honduras | 0,634 | Medium | Francisco Morazan | 0.705 | High |
| Center 06 - Honduras | 0,634 | Medium | La Paz | 0.582 | Medium |
| Center 07 - Guatemala | 0,663 | Medium | Southwest | 0.642 | Medium |
| Center 08 - Dominican Republic | 0,756 | High | Region 0 | 0.776 | High |

* The Next Frontier—Human Development and the Anthropocene: UNDP Human Development Report 2020.

Human Development Report Office 2020. <http://hdr.undp.org/en/content/latest-human-development-index-ranking>
